# Effects of Eplerenone on Blood Pressure and Echocardiographic and Serum Biochemical Variables in Five Healthy Dogs: A Pilot Study

**DOI:** 10.1155/2020/5193856

**Published:** 2020-01-13

**Authors:** Shinji Arita, Noboru Arita, Yoshiaki Hikasa

**Affiliations:** ^1^Arita Sougo Animal Hospital, 1-14-6 Nishi, Hachihonmatsu, Higashihiroshima-shi, Hiroshima 739-0147, Japan; ^2^Laboratory of Veterinary Internal Medicine, Joint Department of Veterinary Medicine, Faculty of Agriculture, Tottori University, 4-101 Koyama-Minami, Tottori 680-8553, Japan

## Abstract

Eplerenone (EP), an aldosterone antagonist, is reported to produce renal and cardiac protective effects in noncanine species. However, there are no detailed reports available on cardiovascular effects of EP in dogs. This study aimed to determine effect of EP on echocardiographic parameters, blood pressures, and biochemical variables in healthy dogs. Five healthy Beagle dogs were randomly divided and repeatedly used in each of 3 dose groups, receiving 2.5, 5, or 10 mg/kg BW EP orally q24 h for 4 wks. Serum biochemical test, blood pressure, and Doppler echocardiography measurements were performed before EP administration and at 1, 2, and 4 weeks after EP administration. Treatment with EP reduced mean blood pressure in a dose-dependent manner and significantly (but in a dose-independent manner) decreased left atrium/aorta ratio, early diastolic transmitral flow, early diastolic transmitral flow/late diastolic transmitral flow, peak velocity of early diastolic transmitral flow/peak velocity of early diastolic mitral annular motion, left ventricle and right ventricle Tei indices, stroke volume, cardiac output, and mid systole myocardial velocity gradient 1 to 4 weeks after administration. Deceleration time of early diastolic transmitral flow significantly increased after EP administration. No significant changes were observed in serum biochemical variables. The results indicated that EP might reduce preload, thereby decreasing left atrial size. In addition, reduction of left ventricular stiffness may have theoretically taken place but this could not be tested using the present study design. It is suggested that EP administration within the dose range used in this study is safe for administration to healthy dogs. Further studies are needed to explore both safety and efficacy, as well as to seek a recommended dose range of EP treatment in client-owned dogs with heart disease.

## 1. Introduction

Aldosterone is a steroid hormone that is synthesized in the adrenal cortex and has a role in the renin-angiotensin–aldosterone system (RAAS). It induces fluid retention via the renal mineralocorticoid receptor by reabsorbing sodium and water ions. Aldosterone has been shown to promote myocardial fibrosis in rats [1]. Moreover, in human medicine, aldosterone contributes to the development of congestive heart failure and cardiovascular disease (i.e., injury of the vascular endothelium and baroreceptor dysfunction) [2, 3]. Studies have demonstrated that the mineralocorticoid receptor is present in the kidneys, cardiomyocytes, and vascular endothelial cells in rabbits [4]. Mineralocorticoid receptors are activated in human heart failure [5, 6], and their activation is involved in organ injury in rats, even with low concentrations of aldosterone in the blood [7]. Therefore, it may be important to prevent both the adverse effects of aldosterone and the activation of the mineralocorticoid receptors for the improvement of heart failure.

Spironolactone, an anti-aldosterone drug, has been reported to reduce the mortality rate in dogs with mitral regurgitation caused by myxomatous mitral valve disease [8] and in cats with cardiac failure due to cardiomyopathy [9]. The aldosterone antagonist eplerenone (EP) is a selective blocking agent of the mineralocorticoid receptor [10]. EP also blocks the nongenomic effects of aldosterone in vascular tissues that are not susceptible to spironolactone [11, 12]. Thus, it is suggested that EP may exert cardiovascular effects that are different from those observed by spironolactone. However, there are no available reports demonstrating the similarities and differences between spironolactone and eplerenone in canine practices.

Treatment with EP exerts renal and cardiac protective effects in rat models of heart failure treated with angiotensin II and Nω-Nitro-L-arginine methyl ester [13] and improves myocardial fibrosis in mice models of hypertension [14]. Furthermore, it ameliorates cardiac hypertrophy, fibrosis, and left ventricular ejection fraction reduction in cardiomyocytes of transgenic mice overexpressing 11beta-hydroxysteroid dehydrogenase type 2 [15]. It also improves vascular endothelium injury in rabbit models of arterial sclerosis [16]. In human medicine, EP has been shown to reduce the incidence of atrial fibrillation [17], duration of hospitalization by heart failure, and incidence of mortality by cardiovascular injuries in patients with heart failure of New York Heart Association classes II and III [18, 19]. EP has also been reported to improve the left ventricular dysfunction in canine models of chronic heart failure by intracoronary microembolizations [20, 21]. In these canine models, however, EP has been used only at a high dose (10 mg/kg; q12 hr) that may trigger side effects. As multidrug combination therapy is currently common for heart failure, it may be important to examine the echocardiographic parameters, blood pressure, and blood biochemical effects of low-dose EP.

To the best of our knowledge, no detailed reports are currently available in the veterinary literature addressing the cardiovascular and serum biochemical effects of lower-dose EP in dogs. The aim of this pilot study was, therefore, to characterize the effects of EP on key echocardiographic parameters, blood pressure, and serum biochemical variables in healthy dogs and determine its potential safety. The hypothesis of this study was that EP may lower blood pressure even at low doses and may reduce left ventricular preload and stiffness.

## 2. Materials and Methods

### 2.1. Animals

Five healthy, adult, neutered, female Beagle dogs (age: 1-2 y; mean weight: 8.0 ± 0.3 kg) were used in this study. The dogs were fed a standard commercial dry food formulated for dogs and were raised in an appropriate animal management facility. The physical and hematological examinations performed before the experiments confirmed that all dogs were healthy. The study protocol was principally based on the Guide for the Care and Use of Laboratory Animals developed by the Institute of Laboratory Animal Research of the National Research Council of Japan and approved by the Animal Research Committee of Tottori University.

### 2.2. Experimental Design and Drug Administration

The five dogs were assigned to receive each of the three treatment groups. The dogs received 2.5, 5, or 10 mg/kg of EP (Selara; 25 to 100 mg tablets, Pfizer, Tokyo, Japan) orally q24 h for 4 weeks. Hereafter, the groups are denoted as EP 2.5, EP 5, and EP 10. Treatments were conducted in the order of EP 2.5, EP 5, and EP 10 in 3 dogs, EP 5, EP 10, and EP 2.5 in 1 dog, and EP 10, EP 2.5, and EP 5 in 1 dog. As it has been reported that the half-life of EP in dogs is 2.21 h, and EP and its metabolites are readily excreted [22], the washout period of EP between the groups was set to be at least 30 days. Hematological and serum biochemical tests, blood pressure measurement, and echocardiography were performed immediately before EP administration (time 0; baseline) and at 1, 2, and 4 weeks after the first administration of each EP dose. EP was administered with breakfast, and venous blood sampling and measurements were performed 2 h later.

### 2.3. Sample Collection and Serum Biochemical Analyses

Sample collection and serum biochemical analyses were performed as reported previously [23]: blood (7.0 mL) was collected from a jugular vein of each dog. An aliquot (1.0 mL) was mixed with ethylene diamine tetra-acetic acid (EDTA) for blood cell count, and another aliquot (1.0 mL) was mixed with heparin for plasma biochemical measurements. Another aliquot (3.0 mL) was mixed with aprotinin-containing EDTA to measure the concentration of atrial natriuretic peptide (ANP). The remaining 2 mL of each sample was transferred to a tube for serum collection to measure the concentration of N-terminal pro-brain natriuretic peptide (NT-proBNP). After centrifugation, the plasma or serum was separated. Blood cell counts were performed using an automatic blood cell analyzer (IDEXX VetAutoread; IDEXX, Tokyo, Japan). The concentrations of blood urea nitrogen, creatinine, total bilirubin, total cholesterol, triglyceride, and inorganic phosphorus, along with the activities of aspartate aminotransferase, alanine aminotransferase, alkaline phosphatase, and creatine phosphokinase, were measured using an automatic biochemistry analyzer (FUJI DRI-CHEM 3500 V; Fuji Film Medical, Tokyo, Japan). The concentrations of plasma sodium, potassium, and chloride were measured using a clinical electrolyte analyzer (FUJI DRI-CHEM 800 V; Fuji Film Medical, Tokyo, Japan). The concentration of serum NT-proBNP was measured using an enzyme-linked immunosorbent assay at IDEXX Laboratories, Tokyo, Japan. The level of plasma ANP was measured using a chemiluminescence enzyme immunoassay at Fukuyama Medical Laboratory (Hiroshima, Japan). Serum and plasma samples for the measurement of NT-proBNP and ANP were stored at −40°C and sent to the aforementioned reference laboratories within one day from the time of collection. Other blood samples were analyzed routinely, immediately after sampling.

### 2.4. Echocardiographic Parameters and Blood Pressure Measurements

Echocardiographic parameters and blood pressure measurements were performed as reported previously [23] as follows. Systolic blood pressure (SBP), diastolic blood pressure (DBP), and mean blood pressure (MBP) were measured by the oscillometric method via a noninvasive blood pressure monitor (Dinamap 8300; Critikon, Tampa, Florida, USA) attached to the tail ridge in the prone position. Transthoracic 2D echocardiography (M-122 mode, pulsed continuous wave) and tissue Doppler echocardiography were performed with dogs in right or left lateral recumbency by using a digital ultrasonography system (Prosound *α*7; Hitachi Aloka Medical, Tokyo, Japan) with a 5 MHz probe. Heart rate (HR) was simultaneously calculated from the preceding R-to-R interval on the electrocardiogram. By using the M-mode method, the left atrium/aorta (LA/Ao) ratio was measured from the left ventricular (LV) outflow tract view in the right parasternal long-axis view with dogs in right lateral recumbency. LV fractional shortening (FS) [24], LV ejection fraction (EF) [24], interventricular septum thickness at end-diastole, left ventricular internal dimension at end-diastole, left ventricular posterior wall thickness at end-diastole, interventricular septum thickness at end-systole, left ventricular internal dimension at end-systole, and left ventricular posterior wall thickness at end-systole were measured in the LV short-axis view. By using pulsed Doppler echocardiography with dogs in left lateral recumbency, the peak velocity of early diastolic transmitral flow (*E*), peak velocity of late transmitral flow (*A*), *E*/*A* ratio, and deceleration time of early diastolic transmitral flow (DT_*E*_) were recorded in the left apical four-chamber view. In the apical five-chamber view, a pulsed-wave sample volume was placed immediately below the aortic valve. The cross-sectional area of the left ventricular outflow tract, aortic peak ejection flow velocity (AEV), and velocity time integral (VTI) were measured from this view, and stroke volume (SV) [25] and cardiac output (CO) were calculated. The CO was calculated as SV × HR. Although HR was calculated based on the RR interval, the average value of the five preceding RR-intervals was used to calculate HR for correction of changes in the RR-interval due to sinus arrhythmia. Furthermore, the time (*a*) from the end of the left ventricular active and late inflow to the initiation of the early and passive reinflow was measured using the left apical four-chamber view. The time (*b*) from the onset to the offset of the LV ejection flow was measured using the apical five-chamber view. The LV Tei index was calculated as (*a* − *b*)/*b* [26]. Likewise, the right ventricle (RV) Tei index was determined from the time (*a*) of the end of the right ventricular tricuspid inflow to the initiation of reinflow in the apical four-chamber view and from the time (*b*) of the onset to the offset of the right ventricular ejection flow in the apical short-axis view [26]. By using tissue Doppler imaging, the mitral annular velocity was recorded based on the medial aspect of the mitral valve annulus from the left apical four-chamber view. The peak velocity of early diastolic mitral annular motion (*E*′) and the peak velocity of the late diastolic mitral annular motion (*A*′) were measured using high frequency tissue Doppler imaging, and the ratio of *E*′ to *A*′ (*E*′/A′) was calculated. Furthermore, the ratio of *E* to *E*′ (*E*/*E*′) was calculated. The endomyocardial and epimyocardial velocities were measured at the posterior wall of the LV in the short-axis view, and the myocardial velocity gradient (MVG) was calculated by dividing the difference (endomyocardial velocity − epimyocardial velocity) of the distance between the two points [27]. The MVG was measured at the mid systole (MVGs), early diastole (MVGe), and atrial systole (MVGa). The measurement of each echocardiographic parameter was repeatedly performed for at least three times, and the averaged value was adopted as representative data. The same investigator (S.A.) performed all measurements and follow-up examinations.

### 2.5. Statistical Analysis

All data obtained were analyzed using commercially available software (StatMate3, ATMS, Tokyo, Japan). One-way analysis of variance was used to examine the time effect within each group and the treatment effect at each time point for serum biochemical, echocardiographic, and blood pressure variables. When a significant difference was detected, Tukey's test was used to compare the means. The dose-dependent relationship was analyzed by a linear regression analysis. The results are expressed as mean ± standard error. The level of significance in all tests was set at *P* < 0.05.

## 3. Results

### 3.1. Echocardiographic and Blood Pressure Variables

**Figure 1:**
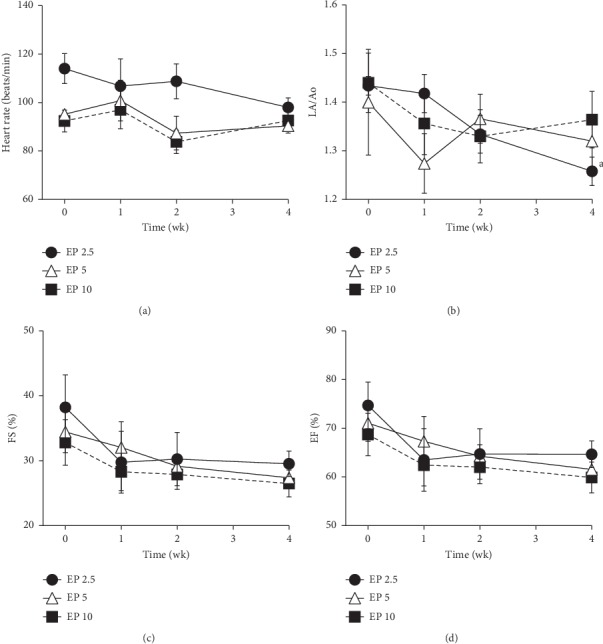
Heart rate (a), left atrium/aorta (LA/Ao) ratio (b), left ventricular fractional shortening (FS) (c), and left ventricular ejection fraction (EF) (d) after eplerenone (EP) administration in dogs. EP 2.5, 5, and 10: eplerenone 2.5, 5, and 10 mg/kg, respectively. Each time point and vertical bar represent the mean and standard error (*n*=5). The lowercase letters indicate a significant difference from baseline (0) value (a—*P* < 0.01).

Figure 1 shows the changes in HR, LA/Ao, FS, and EF. No significant changes were observed in HR after EP administration within or between the groups (Figure 1(a)). At 4 weeks, the LA/Ao decreased significantly in the EP 2.5 group only, compared with that recorded at baseline (Figure 1(b)). Both the FS and EF in all EP-treated groups did not significantly decrease after EP administration (Figures 1(c) and 1(d)). There were no significant differences observed within or between the groups in the left ventricular internal dimensions at both end-diastole and end-systole.

Table 1 shows the changes in *E*, *A*, *E*/*A*, DT_*E*_, and *E*/*E*′ values after EP administration. In the EP 10 group, the *E* values at 1, 2, and 4 weeks after EP administration decreased significantly compared with those observed at baseline. The *A* value did not change significantly in response to any of the EP doses. *E*/*A* in the EP 5 and EP 10 groups decreased significantly compared with baseline date and at 2 weeks and 1 week, respectively. At 2 and 4 weeks, DT_*E*_ in the EP 5 (but not in the EP 10) group increased significantly compared with that recorded at baseline. *E*/*E*′ in the EP 2.5 and EP 10 groups decreased significantly at 2–4 weeks and 1–4 weeks compared with the baseline, respectively. *E*/*E*′ in the EP 5 group did not significantly decrease after EP administration. There were no significant differences between the groups in *E*, *A*, *E*/*A*, DT_*E*_, and *E*/*E*′ values.

**Figure 2:**
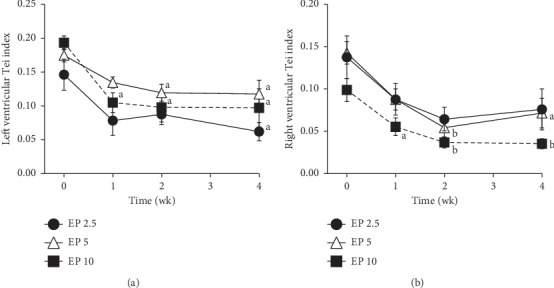
Left (a) and right (b) ventricular Tei indices after eplerenone (EP) administration in dogs. EP 2.5, 5, and 10: eplerenone 2.5, 5, and 10 mg/kg, respectively. Each time point and vertical bar represent the mean and standard error (*n*=5). The lowercase letters indicate a significant difference from baseline (0) value (a—*P* < 0.05; b—*P* < 0.01).

The LV Tei index in the EP 2.5, EP 5, and EP 10 groups decreased significantly at 4 weeks, 2–4 weeks and 1–4 weeks compared with the baseline, respectively (Figure 2(a)). The RV Tei index in the EP 5 and EP 10 groups decreased significantly at 2–4 weeks and 1–4 weeks compared with the baseline, respectively (Figure 2(b)).

Table 2 shows the EP-induced changes in tissue Doppler echocardiographic metrics, including *E*′, *A*′, *E*′/A′, MVGs, MVGe, and MVGa. The *E*′ value in the EP groups did not significantly increase after EP administration. *A*′, *E*′/A′, and MVGa did not change significantly after EP administration in any of the groups. At 4 weeks, MVGs in the EP 2.5 group decreased significantly compared with that observed at baseline. MVGs in the EP 5 and 10 groups and MVGe in all EP-treated groups did not significantly change after EP administration when compared with the baseline. There were no significant differences between the groups in each variable listed in Table 2.

Table 3 shows the changes in AEV, VTI, CO, SV, SBP, DBP, and MBP values after EP administration. The AEV did not significantly decrease after EP administration. Between 1 and 4 weeks, VTI in the EP 5 group decreased significantly when compared with the baseline. VTI in the EP 2.5 and 10 groups also tended to decrease insignificantly after EP administration. CO in the EP 2.5 group decreased significantly at 2–4 weeks when compared with the baseline. SV in the EP 5 and EP 10 groups decreased significantly at 1–4 weeks and 2–4 weeks, respectively. DBP in the EP 5 group decreased significantly at 1 week when compared with that observed at baseline. MBP in EP 5 and EP 10 groups decreased significantly at 1–4 weeks and 4 weeks when compared with that observed at baseline, respectively. SBP at 4 weeks in EP 10 group was significantly lower than that in both EP 2.5 and 5 groups. At 2 weeks, DBP in the EP 5 group was significantly lower than that observed in the EP 2.5 group. MBP at 4 weeks in both EP 5 and 10 groups was significantly lower than that observed in the EP 2.5 group. The EP treatment decreased MBP in a dose-dependent manner (*R*^2^ = 0.4175; *P*=0.0093) at 4 weeks.

No behavioral adverse effects were observed in any of the groups.

### 3.2. Hematological and Blood Biochemical Variables

Table 4 shows the changes in the concentrations of packed cell volume, NT-proBNP, ANP, blood urea nitrogen, creatinine, total protein, sodium, potassium, and chloride. These variables did not change significantly after EP administration in any of the groups (Table 4). Furthermore, white blood cell count and platelet count did not change significantly in any of the groups.

## 4. Discussion

The results of this study revealed that EP administration reduced the LA/Ao, *E* wave, *E*/*A*, and *E*/*E*′ in healthy dogs. A decrease in *E* wave, *E*/*A*, and *E*/*E*′ suggests a decrease in left atrial pressure [28]. A decrease in E/*E*′ is observed with decrease in LA pressure in dogs with mitral valve insufficiency [29]. The changes of these parameters may be due to reduction in preload on LV caused by anti-aldosterone action of EP. The possible EP-induced reduction in LV preload may be responsible for the tendency of decrease in FS, EF, and AEV and significant decrease in VTI, CO, and SV observed in this study. The deduced EP-related mild reduction in blood volume, venous return, and preload is also consistent with the decreased LA/Ao ratio, the decreased *E*, and decreased *E*/*E*′ ratio, with no apparent change in *E*′.

This study revealed that EP administration reduced CO, SV, MVGs, MBP, and DBP and tended to reduce FS and SBP in clinically normal dogs. The decrease in the LV preload is known to induce the reduction of SV via the Frank–Starling mechanism in a healthy heart. The observed decrease in diastolic blood pressure induced by EP may be due to a decrease in either CO or systemic vascular resistance or both [30, 31]. The EP-induced decrease in FS was inconsistent with the results of a previous report showing that EP increased FS in a transgenic mouse model of aldosterone-driven cardiac hypertrophy and heart failure [15]. However, this discrepancy may be due to the species and study design differences (e.g., the present study had no experimentally induced RAAS changes, myocardial hypertrophy, or heart failure). Moreover, an increase rather than a decrease in both preload and afterload via the activation of RAAS, along with heart failure, may have occurred in the experimental mouse study, which is the opposite of what likely occurred in the present healthy dog study [15]. A possible decrease in both systemic vascular resistance and LV afterload could have been caused in the present study's dogs by EP-related dilation of arterial vascular smooth muscle [30, 31]. Therefore, it might be expected that the anti-aldosterone activity and mineralocorticoid receptor blocking effect of EP [30, 31] may reduce not only preload but also afterload, thus leading to improvements in FS in dogs with heart failure. Such clinically relevant effects, however, were beyond the scope of the present study and were not tested.

This study revealed that EP administration significantly increased DT_*E*_ and significantly reduced *E*/*E*'. It has been reported that DT_*E*_ can be prolonged with a left ventricular stiffness decrease [32]. Therefore, it is possible that EP reduces the stiffness of LV, as DT_*E*_ extended even in the face of deduced left ventricular preload reduction. However, LV stiffness, too, was not directly investigated or otherwise quantified in the present study. The changes of these parameters might indirectly suggest the enhancement of LV diastolic function [27, 28, 33]. However, preload reduction alone could have explained our findings even without any improvement in diastolic function via EP-induced anti-aldosterone activity [10]. It has been reported that EP administration attenuated ventricular hypertrophy, ventricular fibrosis, myocardial stiffening, and relaxation abnormality in rats with experimentally induced diastolic heart failure [34]. Although it has been reported that EP increases FS in a transgenic murine model [15], our study found that FS was, in fact, decreased by EP treatment. This might be, at least partially, due to the normal LV function in the present study cohort. FS was likely decreased by the reduction of venous return following EP treatment. The EP-induced decrease in MVGs also might have also been caused by the decrease in LV preload, which reduces the contraction rate of LV cardiomyofibrils. However, these EP effects might be amplified in patients with an activation of the mineralocorticoid receptor and RAAS, which is not thought to have occurred in the present study. On the other hand, as healthy dogs were used in this study, EP-induced LA size and LV stiffness reduction might have been mediated by other mechanisms, including the involvement of nitric oxide: endothelial nitric oxide synthase produces nitric oxide, thus leading to vasorelaxation [35] and myocardial relaxation [36]. EP has been reported to activate endothelial nitric oxide synthase in spontaneous hypertensive rats [37]. Therefore, it is possible that the action of nitric oxide derived from the activation of endothelial nitric oxide synthase is in part involved in echocardiographic effects induced by EP.

In general, a reduction in SV and CO is thought to worsen “forward” heart failure, while in congestive heart failure a reduced preload should contribute more to symptomatic relief than the potential harm brought about by the concomitant reduction of fractional shortening [38]. Our study revealed that both LV and right ventricle Tei indices decreased after EP administration in dogs. The Tei index reflects the comprehensive function of cardiac contractility and dilatability [26]. Therefore, it is conceivable that the cardiac performance is well maintained after EP administration in healthy dogs.

During treatment with EP, patients/animals should be monitored for the development of hyperkalemia [19]. However, in the present study, there were no significant changes in the concentrations of blood urea nitrogen, creatinine, and plasma electrolytes (including potassium) after EP administration. This finding is consistent with previous results on the long-term effects of treatment with high-dose EP in a canine model of ischemic heart failure [20]. Moreover, there were no adverse events observed with regard to other blood variables and clinical symptoms. These findings suggest that EP administration within the dose range used in this study is safe for the treatment of healthy dogs. However, since EP carries the possible effect of reducing both preload and afterload, attention should always be paid to inadvertently triggered dehydration, hypovolemia, or hypotension. In this specific regard, the indirect evidence of EP-related preload reduction without evidence of concomitant prerenal azotemia or hemoconcentration should be viewed as encouraging because it supports the notion that if there was indeed any EP-related volume reduction in these healthy dogs, it was only mild.

There are some limitations in this study. In our pilot study design, only five dogs were used repeatedly, and all were females. Plasma aldosterone concentrations were not measured in this study. In humans, EP has been reported to have equivalent antihypertensive effect with both SID and BID administrations [31]. This study was designed therefore with SID EP administration. As no food effect is shown after oral EP administration was observed in a pharmacokinetic analysis [39], EP tablets were mixed with food in the present study. In addition, due to sound beam to tissue angulation considerations, for more accurate SV and CO data, the measurement of aortic diameter used for calculating the cross-sectional area of the left ventricular outflow tract might be better imaged from the craniodorsal left view rather than from the apical five-chamber view performed in this study.

Similarly, the LA/Ao ratio in the present study was based on M-mode imaging in the right parasternal long-axis view rather than on more widely accepted 2D imaging in the right parasternal short-axis view. Finally, in the present study, the MBP decreased in a dose-dependent manner. While no significant dose-dependent effects of EP were observed for any of the other quantified parameters, some echocardiographic parameters did show significant changes at the low EP dose. It is suggested that EP can initiate cardiovascular effects even at lower doses (2.5 and 5 mg/kg, SID) than those (10 mg/kg, BID) reported previously in dogs [20, 21]. The reasons why those same effects were not exacerbated, or even merely demonstrated, with higher doses remain unclear. This is, in fact, one of the present study's limitations and could actually reflect Type I or Type II errors, resulting from lack of sufficient statistical power.

## 5. Conclusions

To the best of the authors' knowledge, the present pilot study is the first to systematically investigate the effect of EP on key echocardiographic parameters, blood pressure, and serum biochemical variables in healthy dogs. The present study indicated that EP is likely to reduce preload and therefore reduces left atrial size and potentially left ventricular stiffness. The present results also suggest that EP administration within the dose range used in this study is safe when given to healthy dogs. Our findings should be taken into consideration when designing future research on the clinical safety and efficacy of EP in canine cardiovascular disease.

## Figures and Tables

**Table 1:** Peak velocity of early diastolic transmitral flow (*E*), peak velocity of late transmitral flow (*A*), ratio of the peak velocity of early diastolic transmitral flow to the peak velocity of late transmitral flow (*E*/*A*), deceleration time of early diastolic transmitral flow (DT_*E*_), and ratio of the peak velocity of early diastolic transmitral flow to peak the velocity of early diastolic septal mitral annular motion (*E*/*E*′) values after eplerenone (EP) administration in dogs.

Variables	Group	Time after EP administration (weeks)			
		0	1	2	4
*E* (cm/s)	EP 2.5	75.8 ± 5.8	66.6 ± 7.3	66.6 ± 3.1	64.1 ± 4.2
	EP 5	64.9 ± 2.3	60.7 ± 3.0	59.4 ± 2.4	60.3 ± 1.6
	EP 10	73.5 ± 3.2	58.0 ± 3.0^b^	61.1 ± 2.1^a^	58.0 ± 1.7^b^

*A* (cm/s)	EP 2.5	50.4 ± 3.8	51.3 ± 8.9	45.5 ± 3.7	46.1 ± 5.0
	EP 5	36.3 ± 2.6	40.2 ± 1.7	44.8 ± 4.2	39.7 ± 3.0
	EP 10	42.7 ± 1.7	43.5 ± 2.8	42.2 ± 2.7	36.5 ± 2.2

*E*/*A*	EP 2.5	1.51 ± 0.06	1.38 ± 0.13	1.49 ± 0.08	1.44 ± 0.13
	EP 5	1.82 ± 0.14	1.52 ± 0.09	1.35 ± 0.09^a^	1.54 ± 0.08
	EP 10	1.72 ± 0.03	1.34 ± 0.08^a^	1.47 ± 0.11	1.60 ± 0.06

DT_*E*_ (ms)	EP 2.5	85.4 ± 2.9	111.6 ± 8.2	106.8 ± 5.8	103.2 ± 7.9
	EP 5	78.0 ± 5.4	97.2 ± 3.5	105.6 ± 8.2^a^	111.6 ± 8.6^a^
	EP 10	76.8 ± 6.7	102.0 ± 6.0	102.0 ± 11.7	104.4 ± 10.1

*E*/*E*′	EP 2.5	9.4 ± 0.5	7.7 ± 0.6	7.0 ± 0.1^b^	7.6 ± 0.4^a^
	EP 5	8.0 ± 0.4	6.8 ± 0.4	6.9 ± 0.3	7.6 ± 0.3
	EP 10	9.8 ± 0.5	7.0 ± 0.6^b^	7.2 ± 0.6^b^	7.3 ± 0.3^a^

Each value represents the mean ± standard error (*n*=5). EP 2.5, 5, and 10: eplerenone 2.5, 5, and 10 mg/kg, respectively. ^a,b^Value differs significantly (^a^*P* < 0.05; ^b^*P* < 0.01) from the baseline (time 0) value.

**Table 2:** Peak velocity of early diastolic septal mitral annular motion (*E*′), peak velocity of diastolic septal mitral annular motion (*A*′), ratio of the peak velocity of early diastolic mitral annular motion to the peak velocity of diastolic mitral annular motion (*E*′/*A*′), mid systolic myocardial velocity gradient (MVGs), early diastolic myocardial velocity gradient (MVGe), and atrial systolic myocardial velocity gradient (MVGa) after eplerenone (EP) administration in dogs.

Variables	Group	Time after EP administration (weeks)			
		0	1	2	4
*E*′ (cm/s)	EP 2.5	8.1 ± 0.4	8.6 ± 0.6	9.5 ± 0.5	8.5 ± 0.4
	EP 5	8.1 ± 0.3	9.0 ± 0.4	8.7 ± 0.5	8.0 ± 0.4
	EP 10	7.6 ± 0.6	8.4 ± 0.7	8.7 ± 0.8	8.1 ± 0.5

*A*′ (cm/s)	EP 2.5	5.7 ± 0.4	5.9 ± 0.3	6.3 ± 0.5	5.3 ± 0.3
	EP 5	5.1 ± 0.3	5.9 ± 0.4	5.5 ± 0.3	5.2 ± 0.4
	EP 10	5.0 ± 0.3	6.1 ± 0.4	5.7 ± 0.3	4.9 ± 0.2

*E*′/A′	EP 2.5	1.44 ± 0.09	1.46 ± 0.09	1.53 ± 0.11	1.59 ± 0.08
	EP 5	1.63 ± 0.10	1.55 ± 0.10	1.58 ± 0.12	1.57 ± 0.14
	EP 10	1.50 ± 0.08	1.38 ± 0.08	1.53 ± 0.11	1.66 ± 0.07

MVGs (s)	EP 2.5	2.61 ± 0.17	2.04 ± 0.19	1.94 ± 0.30	1.72 ± 0.18^a^
	EP 5	2.38 ± 0.34	1.79 ± 0.28	1.86 ± 0.29	1.56 ± 0.18
	EP 10	1.94 ± 0.13	1.71 ± 0.23	1.77 ± 0.27	1.60 ± 0.25

MVGe (s)	EP 2.5	−1.74 ± 0.34	−2.38 ± 0.61	−2.47 ± 0.58	−2.54 ± 0.58
	EP 5	−1.44 ± 0.19	−1.62 ± 1.00	−2.44 ± 0.56	−2.48 ± 0.43
	EP 10	−1.71 ± 0.26	−2.21 ± 0.26	−2.58 ± 0.20	−2.24 ± 0.38

MVGa (s)	EP 2.5	−1.19 ± 0.24	−0.36 ± 0.92	−0.64 ± 0.51	−1.01 ± 0.47
	EP 5	−0.58 ± 0.73	−1.45 ± 0.30	−0.69 ± 0.58	−0.60 ± 0.43
	EP 10	−0.93 ± 0.16	−1.01 ± 0.17	−1.24 ± 0.17	−1.34 ± 0.34

Each value represents the mean ± standard error (*n*=5). EP 2.5, 5, and 10: eplerenone 2.5, 5, and 10 mg/kg, respectively. ^a^Value differs significantly from the baseline (time 0) value at *P* < 0.05.

**Table 3:** Aortic ejection flow velocity (AEV), velocity time integral (VTI), stroke volume (SV), cardiac output (CO), systolic blood pressure (SBP), diastolic blood pressure (DBP), and mean blood pressure (MBP) values after eplerenone (EP) administration in dogs.

Variables	Group	Time after EP administration (weeks)			
		0	1	2	4
AEV (cm/s)	EP 2.5	81.4 ± 5.4	68.3 ± 5.7	73.3 ± 3.3	76.2 ± 3.4
	EP 5	77.7 ± 4.9	68.7 ± 4.0	66.1 ± 2.6	66.7 ± 3.0
	EP 10	71.3 ± 3.3	67.5 ± 2.6	67.3 ± 4.3	67.4 ± 2.7

VTI (cm)	EP 2.5	9.0 ± 0.6	7.6 ± 0.6	7.8 ± 0.4	7.9 ± 0.4
	EP 5	9.4 ± 0.4	7.6 ± 0.3^a^	7.8 ± 0.4^a^	7.6 ± 0.4^a^
	EP 10	8.7 ± 0.1	7.7 ± 0.2	7.8 ± 0.5	7.4 ± 0.5

SV (mL)	EP 2.5	9.1 ± 0.5	7.8 ± 0.5	7.8 ± 0.6	7.5 ± 0.3
	EP 5	9.3 ± 0.4	7.2 ± 0.3^b^	7.6 ± 0.3^a^	7.5 ± 0.4^a^
	EP 10	9.2 ± 0.5	7.9 ± 0.2	7.7 ± 0.3^a^	7.4 ± 0.4^a^

CO (L/min)	EP 2.5	1.13 ± 0.14	0.84 ± 0.11	0.74 ± 0.07^a^	0.65 ± 0.03^a^
	EP 5	0.87 ± 0.11	0.67 ± 0.07	0.64 ± 0.06	0.65 ± 0.05
	EP 10	0.79 ± 0.08	0.72 ± 0.06	0.66 ± 0.04	0.60 ± 0.04

SBP (mmHg)	EP 2.5	121 ± 3.6	117 ± 4.3	111 ± 4.0	114 ± 4.8
	EP 5	123 ± 5.0	113 ± 4.5	113 ± 5.5	111 ± 5.2
	EP 10	119 ± 7.6	108 ± 6.3	105 ± 4.6	96 ± 4.4^cd^

DBP (mmHg)	EP 2.5	74 ± 2.5	65 ± 2.7	80 ± 7.8	72 ± 5.8
	EP 5	71 ± 4.7	55 ± 4.0^a^	60 ± 3.6^c^	60 ± 2.2
	EP 10	71 ± 3.3	59 ± 6.5	61 ± 3.0	60 ± 3.6

MBP (mmHg)	EP 2.5	94 ± 2.5	85 ± 4.1	88 ± 6.9	89 ± 4.9
	EP 5	92 ± 2.6	73 ± 4.4^a^	78 ± 4.9	76 ± 2.9^ac^
	EP 10	89 ± 3.3	76 ± 5.2	77 ± 3.7	72 ± 2.1^ac^

Each value represents the mean ± standard error (*n*=5). EP 2.5, 5, and 10: eplerenone 2.5, 5, and 10 mg/kg, respectively. ^a,b^Value differs significantly (^a^*P* < 0.05; ^b^*P* < 0.01) from the baseline (time 0) value. ^c^Value differs significantly from the EP 2.5 group at *P* < 0.05. ^d^Value differs significantly from the EP 5 group at *P* < 0.05.

**Table 4:** Blood paced cell volume (PCV), serum N-terminal pro-brain natriuretic peptide (NT-pro BNP), plasma atrial natriuretic peptide (ANP), blood urea nitrogen, creatinine, total protein, sodium, potassium, and chloride after eplerenone (EP) administration in dogs.

Variables	Group	Time after EP administration (weeks)			
		0	1	2	4
PCV (%)	EP 2.5	46.1 ± 1.3	43.8 ± 1.5	45.3 ± 1.9	45.0 ± 1.5
	EP 5	45.6 ± 2.7	44.2 ± 2.0	45.2 ± 3.0	45.3 ± 3.1
	EP 10	44.4 ± 2.6	45.7 ± 2.6	44.8 ± 2.3	43.9 ± 2.3

NT-pro BNP (pmoL/L)	EP 2.5	180 ± 53	212 ± 44	277 ± 72	186 ± 75
	EP 5	404 ± 105	228 ± 68	327 ± 87	404 ± 81
	EP 10	209 ± 72	266 ± 42	277 ± 53	224 ± 48

ANP (pg/mL)	EP 2.5	41.3 ± 6.1	31.1 ± 5.9	39.0 ± 8.2	29.5 ± 6.3
	EP 5	44.6 ± 12.5	42.8 ± 12.8	33.8 ± 6.8	29.5 ± 4.8
	EP 10	40.1 ± 12.7	24.4 ± 3.4	27.6 ± 6.2	25.6 ± 2.7

Blood urea nitrogen (mg/dL)	EP 2.5	16.3 ± 1.2	17.3 ± 0.7	14.0 ± 1.0	19.4 ± 1.4
	EP 5	14.7 ± 0.8	16.2 ± 1.4	17.4 ± 2.3	16.4 ± 1.9
	EP 10	15.5 ± 1.3	14.7 ± 0.8	14.8 ± 1.2	12.9 ± 0.8

Creatinine (mg/dL)	EP 2.5	0.7 ± 0.1	0.6 ± 0.1	0.6 ± 0.1	0.7 ± 0.1
	EP 5	0.6 ± 0.1	0.6 ± 0.1	0.7 ± 0.1	0.7 ± 0.1
	EP 10	0.7 ± 0.1	0.6 ± 0.1	0.7 ± 0.1	0.6 ± 0.1

Total protein (g/dL)	EP 2.5	6.0 ± 0.1	6.1 ± 0.1	6.1 ± 0.1	6.2 ± 0.1
	EP 5	6.0 ± 0.1	6.1 ± 0.2	6.1 ± 0.1	6.2 ± 0.1
	EP 10	6.1 ± 0.1	6.2 ± 0.1	6.3 ± 0.2	6.3 ± 0.1

Sodium (mmoL/L)	EP 2.5	145 ± 0.2	145 ± 0.4	146 ± 0.6	146 ± 0.8
	EP 5	146 ± 0.2	144 ± 0.8	146 ± 0.5	144 ± 1.4
	EP 10	144 ± 0.7	145 ± 0.7	145 ± 1.1	144 ± 0.4

Potassium (mmoL/L)	EP 2.5	4.4 ± 0.2	4.3 ± 0.2	4.4 ± 0.1	4.2 ± 0.1
	EP 5	4.2 ± 0.2	4.1 ± 0.1	4.0 ± 0.2	4.0 ± 0.2
	EP 10	4.2 ± 0.1	4.2 ± 0.1	4.0 ± 0.1	4.2 ± 0.1

Chloride (mmoL/L)	EP 2.5	115 ± 1.4	116 ± 1.1	116 ± 0.8	116 ± 1.6
	EP 5	118 ± 0.7	116 ± 0.7	116 ± 0.9	117 ± 0.9
	EP 10	116 ± 1.0	114 ± 1.6	116 ± 1.7	115 ± 1.3

Each value represents the mean ± standard error (*n*=5). EP 2.5, 5, and 10: eplerenone 2.5, 5, and 10 mg/kg, respectively.

## Data Availability

The raw data used to support the findings of this study are available from the corresponding author upon request.
